# Identification of *SSTR5* Gene Polymorphisms and Their Association With Growth Traits in Hulun Buir Sheep

**DOI:** 10.3389/fgene.2022.831599

**Published:** 2022-04-26

**Authors:** Xue Li, Ning Ding, Zhichao Zhang, Dehong Tian, Buying Han, Dehui Liu, Sijia Liu, Fei Tian, Dejun Fu, Xiaoliang Song, Kai Zhao

**Affiliations:** ^1^ Key Laboratory of Adaptation and Evolution of Plateau Biota, Qinghai Provincial Key Laboratory of Animal Ecological Genomics, Northwest Institute of Plateau Biology, Chinese Academy of Sciences, Xining, China; ^2^ University of Chinese Academy of Sciences, Beijing, China; ^3^ Inner Mongolia Daxing ‘anling Agricultural Reclamation Group Co. LTD., Hulun Buir, China

**Keywords:** SSTR5, association, growth traits, Hulun Buir sheep, haplotypes

## Abstract

The aim of this study was to locate *SSTR5* polymorphisms and evaluate their association with growth traits in Hulun Buir sheep. The study followed up 884 Hulun Buir sheep from birth to 16 months of age, which were born in the same pasture and the same year, and a consistent grazing management strategy was maintained. The birth weight (BRW) was recorded at birth, and body weight (BW), body height (BH), body length (BL), chest circumference (ChC), chest depth (ChD), chest width (ChW), hip width (HW), and cannon circumference (CaC) were measured at 4 and 9 months of age. BW, BH, BL, ChD, HW, and CaC were also recorded at 16 months of age. Based on the growth traits, 233 sheep were selected as experimental animals. Sanger sequencing was performed, and seven single-nucleotide polymorphisms (SNPs) were identified. Association analyses of the SNPs and the growth traits were then conducted. Seven SNPs of the *SSTR5* exhibited moderate polymorphism (0.25<PIC<0.5) and were consistent with the Hardy–Weinberg equilibrium. SNP7 (T989C, rs601836309) caused a change in amino acid sequences, while others did not cause any change. The genotypes of SNP1 (C186T, s400914340) were significantly associated with BW, ChW, and ChC at 4 months of age and with HW at 9 months of age (*p*<0.05). These genotypes also showed extremely significant association with CaC at 4 months of age (*p*<0.01). The genotypes of SNP7 exhibited a significant association with ChW and CaC at 4 and 9 months of age, respectively. Moreover, the genotypes of SNP3 (T384C, rs413380618)) and SNP4 (T537C, rs605867745) were significantly associated with CaC at 9 months of age (*p*<0.05). Linkage disequilibrium was observed among the seven SNPs with five haplotypes. However, these haplotypes were not associated with growth traits at different ages. In conclusion, SNP1, SNP3, SNP4, and SNP7 may serve as molecular markers for the growth traits of Hulun Buir sheep.

## 1 Introduction

Hulun Buir sheep is an esteemed local mutton breed in Hulun Buir, Inner Mongolia, China. This breed exhibits outstanding stress resistance, strong adaptability, stable heredity and provides high-quality, low-fat meat with a variety of amino acids. As a traditional mutton sheep breed, Hulun Buir sheep are not selected via advanced breeding methods; therefore, the breed exhibits low productivity, a slow growth rate, and a low slaughter rate. Many candidate genes have been reported to regulate metabolism and control the growth rate of domestic animals (Al-Mamun et al., 2015; Wang et al., 2015; La et al., 2019). Genetic variations in the candidate genes have been widely used as molecular markers, accelerating the breeding process and improving productivity. For example, new breeds of beef cattle with a myostatin mutation have been established; this molecular marker is also used in pig breeding (Cyranoski, 2015). Additionally, the ovine *VRTN* gene may be a new candidate gene for breeding sheep with more thoracic vertebrae (Li C et al., 2019).

In our previous experiments on the liver transcriptome of Hulun Buir sheep with different growth traits, somatostatin receptor subtype 5 (*SSTR5*) was identified as a differentially expressed gene. In the present study, we explored whether this gene would affect the growth traits of Hulun Buir sheep.

As a somatostatin (*SST*) receptor, *SSTR5* has functions inseparable from those of *SST*. *SST*, also known as growth hormone (GH)-inhibiting hormone or somatotropin release-inhibiting factor, is considered as a hypothalamic factor that inhibits the secretion of GH (Brazeau et al., 1973). In mammals, there are five somatostatin receptor subtypes (SSTR5). SST and SSTR are widely distributed in the central nervous system, pancreas, intestines, stomach, kidney, liver, pancreas, lungs, and placenta and has a variety of biological functions (Finley et al., 1981; Maecke and Reubi, 2011; Quan et al., 2020). SST employs diverse mechanisms to regulate growth, but its activity depends on the binding of G-protein-coupled somatostatin receptors (Anzola et al., 2019). In addition to exerting an inhibitory effect on GH release (Luque et al., 2006), SSTR also represses the secretion of prolactin, thyroid-stimulating hormone (TSH) (Colturi et al., 1984), stomach hormones, GH-releasing hormone (GHRH), secretin, glucagon, insulin, and SST in the pancreas (Lloyd et al., 1997). Furthermore, SSTR decreases the nutrient absorption rate in the gastrointestinal tract by inhibiting the secretion of gastrointestinal hormones and digestive enzymes (Tulassay, 1998). In addition, SSTR controls digestion and absorption rates by reducing gastrointestinal motility, gallbladder contraction, and blood flow, which negatively affects feed conversion and growth characteristics (Strowski et al., 2000).

The nucleotide sequence of *SSTR5* is highly conserved among species; ovine *SSTR5* shares 85% sequence homology with humans and rats and 87% homology with mice. The ovine *SSTR5* transcript has four exons and three introns (ENSOARG00000014478.1) located on chromosome 24 (GenBank, Gene ID: 443,210), encoding 1,044-bp base (rs 812,728–813866) and 347-amino acid residues. As a receptor for somatostatin, SSTR5 plays an important role in many physiological processes, such as GH release, cell anti-proliferation, and regulating a variety of signal transduction pathways (Cattaneo et al., 1996; Melmed, 2003). *SSTR5* is also one of the major *SSTRs* expressed in the islets of Langerhans and plays an essential role in mediating the inhibitory effect of SST on insulin expression, secretion, and cell proliferation (Fagan et al., 1998).

Based on our previous experiments, and considering the importance of *SSTR5* in controlling the growth hormone axis and the lack of research on the effect of the *SSTR5* gene on growth traits in sheep, we conducted a molecular characterization of *SSTR5*, identified polymorphisms, and analyzed associations between different genotypes and growth traits in Hulun Buir sheep. The current study generated novel information about the genetic resources of Hulun Buir sheep, serving as a foundation for future applications of molecular markers in breeding.

## 2 Materials and Methods

### 2.1 Experimental Animals and Growth Trait Data Acquisition

The study included 884 Hulun Buir sheep, the progeny of unrelated rams, born in the same pasture in March 2019 at the Hulun Buir Sheep Breeding Farm in Hulun Buir, Inner Mongolia, China. The growth traits of each sheep were recorded from birth to adulthood (16 months of age). During the experiment, all sheep were allowed to graze freely on natural pasture and had free access to water.

At birth, only birth weight (BRW) was recorded. Body weight (BW), body length (BL), body height (BH), chest circumference (ChC), chest depth (ChD), chest width (ChW), hip width (HW), and cannon circumference (CaC) were recorded at 4 and 9 months of age. BW, BL, BH, ChD, HW, and CaC were again recorded at 16 months of age. Size and weight were measured using a veterinary measuring tape and a sensitive platform balance, respectively (Zhang et al., 2016).

Based on the growth traits, a total of 233 healthy, disease-free sheep were selected as experimental animals, comprising 119 sheep with the fastest growth rate and 114 sheep with the slowest growth rate (124 females and 109 males). There were significant differences in growth traits between the two extreme populations (Supplementary Table S1). All animal experiments were conducted following the procedures described in the “Guidelines for animal care and use” manually approved by the Animal Care and Use Committee, Northwest Institute of Plateau Biology, Chinese Academy of Sciences (NWIPB2020302, 13 April 2020).

### 2.2 Primer Design and Sequencing

A 0.5-cm^2^ ear tissue sample was collected from each sheep at birth for DNA extraction and preserved in 75% alcohol. DNA was purified using a DNA extraction kit (TIANGEN, Beijing, China), and the quality was evaluated by running samples on a gel. Primers were designed for all exons of the *SSTR5* using Primer3 v0.4.0 (1) (Koressaar and Remm, 2007). The *SSTR5* transcript has four exons (ENSOARG00000014478.1); one primer pair was designed to cover exons 1 to 2 and another to cover exons 3 and 4. Information about primers is presented in Table 1.

**TABLE 1:** Primer information of *SSTR5* of Hulun Buir sheep.

Primer name	Primer sequences (5–3′)	Size (bp)	Tm (°C)
E1-2	F: CCTCGGCTCAGTCGCTC	761	60
	R: TAG​CAC​AGG​CAG​ATG​ACC​AG		
E3-4	F: TGG​AAC​ACC​TGC​AAC​CTC​AG	759	60
	R: GTC​TCC​TCT​TCT​GCT​CCA​GC		

PCR amplifications were performed in a 30 μl reaction volume consisting of 1.0 μl of DNA, 15 μl of 2×Taq PCR Master Mix (Sangon, Shanghai, China), 1.0 μl of each primer, and double-distilled water (dH_2_O) to make up the volume. Amplifications were performed using Bio-Rad S1000 thermal cyclers (Bio-Rad, Hercules, CA, United States). The thermal profile was as follows: initial denaturation at 94°C for 2 min, followed by 35 cycles at 94°C for 10 s (denaturation), 60°C for 30 s (annealing), 72°C for 60 s (elongation), with a final extension step at 72°C for 5 min. The PCR products were visualized using 1.0% agarose gel electrophoresis to determine amplicon quality and quantity. The sequencing was performed using Sanger sequencing (Agilent 3,730, United States). Sequence alignment and SNP identification were conducted via MEGA (version 5.0) (Electronics Ltd., Kuopio, Finland). DNAMAN software (version 5.2.10) (Lynnon BioSoft, Vaudreuil, Canada) was used to conduct sequence analyses.

### 2.3 Bioinformatics Analysis of Non-Synonymous Mutations

Protein analyses were conducted with ExPASy tools (http://expasy.org/tools/), and parameters including molecular weight, isoelectric point, instability index, aliphatic index, and grand average of hydropathicity were computed. SignalP 4.0 (http://www.cbs.dtu.dk/services/SignalP/) was used to predict the presence of signal peptides. NetOGlyc 3.1 (http://www. cbs. dtu.dk/services/NetOGlyc/) and NetNGlyc 1.0 (http://www.cbs. dtu. dk/services/NetNGlyc/) were used to predict potential O– and N–glycosylation sites, respectively. NetPhos2.0 (http://www.cbs.dtu.dk/services/NetPhos/) was used to predict phosphorylation sites.

### 2.4 Population Genetic Analyses

Population genetic indices including allele frequency, heterozygosity (He), observed heterozygosity (Ho), effective allele numbers (Ne), and the polymorphism information content (PIC) were analyzed as previously reported (Nei and Roychoudhury, 1974). Genotypes of SNPs were tested for the Hardy–Weinberg equilibrium (HWE) (Ortega et al., 2016). Linkage disequilibrium (LD) and haplotypes analysis were conducted using Haploview (v.4.2) (Barrett et al., 2005).

### 2.5 Statistical Analysis

Measured traits were tested for normality by using the Shapiro–Wilk test in SPSS Statistics (V.19, IBM, Armonk, NY, United States). Pearson’s correlation coefficients were calculated to determine the correlation among the following measured traits at 4, 9, and 16 months of age: BW, BL, BH, ChC, ChD, ChW, HW, and CaC. SPSS was used to perform all analyses, and values are expressed as mean ± standard error. General linear mixed models were established to examine the associations between the genotypes and individual growth traits, and statistical significance was defined at *p*<0.05. In this model, genotype and gender were fixed factors, and their interaction was tested. If an interaction between genotype and gender was identified, the following statistical model was used:

Y = μ+ Genotype + Gender + Combination + ε, where Y is the trait measured for each animal (BW, BL, BH, ChW, ChD, ChW, HW, and CaC), μ is the mean value of Y, Genotype is the genotype effect, Gender is the gender effect, Combination is the combined effect of the gender and genotype, and ε is the random error, assumed to be independent and normally distributed; N (0, σ2). If no interaction between genotype and gender was identified, the following statistical model was used: Y = μ + Genotype + ε, where Y is the trait measured for each animal (BW, BL, BH, ChW, ChD, ChW, HW, and CaC), μ is the mean value of Y, Genotype is the genotype effect, and ε is the random error, assumed to be independent and normally distributed; N (0, σ2).

## 3 Results

### 3.1 Correlations Between Growth Traits

All data conformed to a normal distribution, indicating suitability for subsequent analyses. At 4 months of age, BW exhibited strong correlations (|r| > 0.7) with BL, ChW, and ChD; BL exhibited strong correlations with ChW and ChD; BH exhibited a strong correlation with ChD; ChD exhibited a strong correlation with HW. At 9 months, BW exhibited strong correlations with BL, BH, ChC, and HW; BL exhibited a strong correlation with BH; BH exhibited a strong correlation with ChC; and ChC exhibited a strong correlation with HW. At 16 months, BW exhibited a negligible correlation (|r| ≤ 0.3) with BH, BL exhibited negligible correlations with CaC and ChD, and HW exhibited a negligible correlation with CaC. Moderate correlations (0.3 < |r| ≤ 0.7) were observed among the other traits (Table 2).

**TABLE 2:** Correlations between growth traits of Hulun Buir sheep ^a^.

	BW	BL	BH	ChW	ChD	ChC	HW
4 months of age							
BL	**0.782** ^**^						
BH	0.537^**^	0.512^**^					
ChW	**0.736** ^**^	**0.724** ^**^	0.368^**^				
ChD	**0.803** ^**^	**0.729** ^**^	**0.756** ^**^	0.689^**^			
ChC	0.549^**^	0.510^**^	0.631^**^	0.454^**^	0.671^**^		
HW	0.611^**^	0.538^**^	0.653^**^	0.475^**^	**0.759** ^**^	0.521^**^	
CaC	0.447^**^	0.358^**^	0.331^**^	0.411^**^	0.495^**^	0.348^**^	0.456^**^
9 months of age							
BL	**0.757** ^**^						
BH	**0.855** ^**^	**0.727** ^**^					
ChW	0.647^**^	0.612^**^	0.568^**^				
ChD	0.663^**^	0.654^**^	0.605^**^	0.431^**^			
ChC	**0.863** ^**^	0.672^**^	**0.742** ^**^	0.620^**^	0.648^**^		
HW	**0.742** ^**^	0.561^**^	0.678^**^	0.336^**^	0.560^**^	**0.722** ^**^	
CaC	0.697^**^	0.545^**^	0.642^**^	0.602^**^	0.526^**^	0.565^**^	0.425^**^
16 months of age							
BL	0.479^**^						
BH	0.184^*^	0.346^**^					
ChD	0.343^**^	0.173^*^	0.363^**^				
HW	0.356^**^	0.381^**^	0.581^**^	0.532^**^			
CaC	0.498^**^	0.046	−0.424^**^	0.310^**^			−0.136

BW= body weight; BL= body length; BH= body height; ChW = chest width; ChD = chest depth; ChC = chest circumference; HW= hip width; CaC = cannon circumference.

Correlations with |r| > 0.7 are in bold, *p < 0.05, **p < 0.01.

a Data represent means ± SEM (n = 233).

### 3.2 Polymorphism in *SSTR5*

**FIGURE 1:**
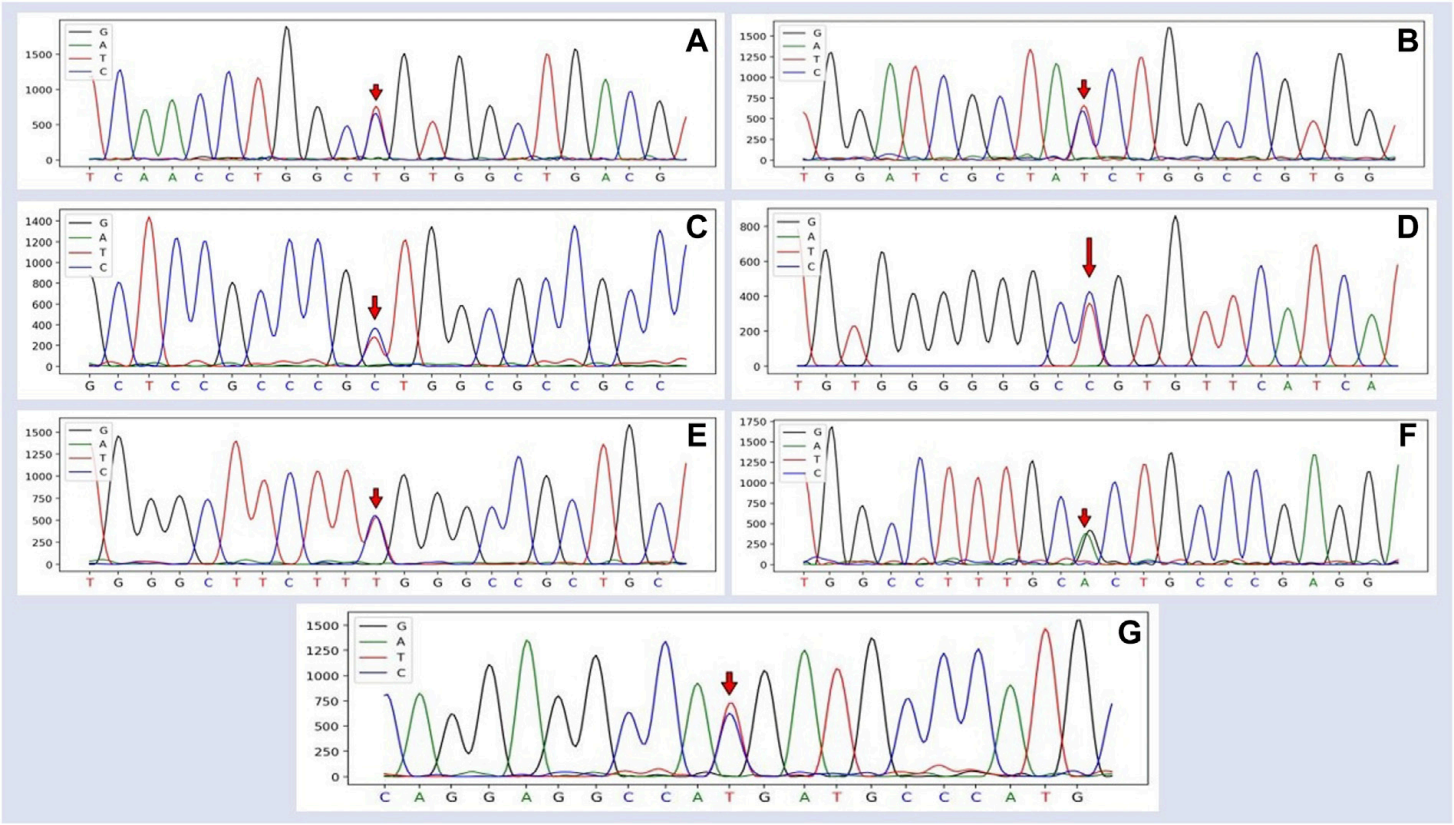
Sequencing peak map of the *SSTR5* and the mutated SNP1–7 site of Hulun Buir sheep. The sequences were analyzed using DNAMAN software. **(A)** The site marked by the red arrow was the SNP1 mutation, which was found and identified in exon 2 of *SSTR5*, C186T (rs400944340). **(B)** The site marked by the red arrow was the SNP2 mutation, which was found and identified in exon 2 of *SSTR5*, C351T (rs404123088). **(C)**The site marked by the red arrow was the SNP3 mutation, which was found and identified in exon 2 of *SSTR5*, T384C (rs413380618). **(D)** The site marked by the red arrow was the SNP4 mutation, which was found and identified in exon 2 of *SSTR5*, T537C (rs605867745). **(E)** The site marked by the red arrow was the SNP5 mutation, which was found and identified in exon 3 of *SSTR5*, C576T (rs593868112). **(F)** The site marked by the red arrow was the SNP6 mutation, which was found and identified in exon 3 of *SSTR5*, G768A (rs403055255). **(G)** The site marked by the red arrow was the SNP7 mutation, which was found and identified in exon 4 of *SSTR5*, T989C (rs601836309).

Seven SNPs were identified by sequencing: C186T (rs400914340), C351T (rs404123088), T384C (rs413380618), T537C (rs605867745), C576T (rs593868112), G768A (rs403055255), and T989C (rs601836309). The first four SNPs were located in exon 2, SNP5 and SNP6 in exon 3, and SNP7 in exon 4. The genetic map of the mutated sites in *SSTR5* based on the sequencing results is illustrated in Figure 1.

### 3.3 Analysis of Physicochemical Properties of Protein With Non-Synonymous Mutations

SNP1–6 were synonymous mutations. SNP7 was a non-synonymous mutation that caused the amino acid at the 330 position to change from methionine (Met) to threonine (Thr). According to analyses of the physicochemical properties of the mutant and wild-type proteins, the molecular weight of the wild-type was less than that of the mutant, and the wild-type had two O-glycosylation sites, but the mutant had only one (Table 3).

**TABLE 3:** Physicochemical properties of protein between wild type and mutant type in SNP7.

Characteristic	Wild type	Mutant type
Number of amino acids	347	347
Molecular weight (kDa)	37828.63	37858.71
Theoretical isoelectric point	9.44	9.44
Instability index	47.68	47.60
Aliphatic index	107.55	107.55
Grand average of hydropathicity	0.532	0.539
Signal peptide	0.2088	0.2088
O-glycosylation site	2	1
N-glycosylation site	3	3
Phosphorylation site	46	46

### 3.4 Population Genetics and the Linkage Disequilibrium Analysis

**FIGURE 2:**
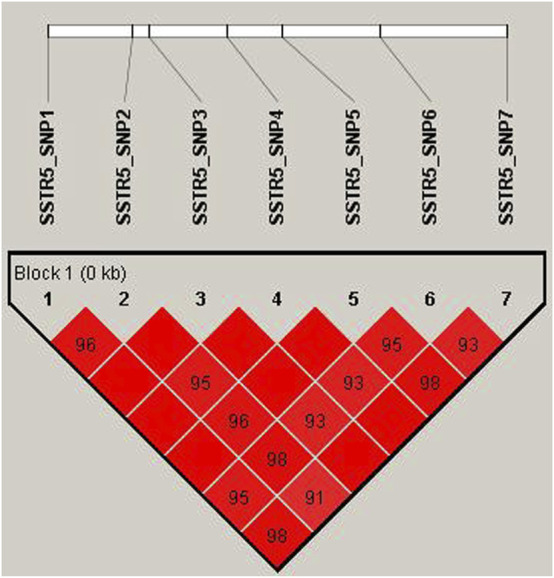
Linkage disequilibrium plot of SNPs in *SSTR5* of in Hulun Buir sheep. The linkage disequilibrium (LD) was estimated among *SSTR5* variations in Hulun Buir sheep. The *R* ^2^ values indicated the group of the SNP1, SNP2, SNP3, SNP4, SNP5, SNP6, and SNP7.

The Ne (effective allele numbers), calculated for each SNP, ranged from 1 to 2. The allele frequency of SNPs was in Hardy–Weinberg equilibrium (*p*<0.05). Based on the PIC, SNP1–7 were classified as moderate polymorphic loci (Table 4). LD analysis revealed a strong LD (D’<0.85) among SNP1–7 (Figure 2), and five common haplotypes were identified in this LD region.

**TABLE 4:** Population genetics analyses of *SSTR5* of in Hulun Buir sheep^a^

SNP	Gene frequency		Ho	He	PIC	Ne	HW
	A	B					
SNP1 (C/T)	0.2756	0.7244	0.3993	0.3991	0.3196	1.6648	0.5888
SNP2 (C/T)	0.6197	0.3803	0.4714	0.4807	0.3603	1.8917	0.6642
SNP3 (T/C)	0.2035	0.7965	0.3241	0.3391	0.2716	1.4796	0.5052
SNP4 (T/C)	0.2137	0.7863	0.3360	0.3519	0.2796	1.5061	0.5188
SNP5 (C/T)	0.2756	0.7244	0.3993	0.3991	0.3196	1.6648	0.5888
SNP6 (G/A)	0.6923	0.3077	0.4260	0.4893	0.3353	1.7423	0.6172
SNP7 (T/C)	0.2158	0.7842	0.3385	0.3562	0.2812	1.5117	0.5216

He = heterozygosity; Ho = homozygosity; PIC= polymorphism information content; Ne = effective allele numbers; HW= Hardy–Weinberg equilibrium.

a Group size of population genetics analyses was *n* = 233.

### 3.5 Association Analysis of Genetic Variants and Haplotypes in *SSTR5* With Growth Traits of Hulun Buir Sheep

The results of association analyses of the *SSTR5* SNP genotypes and the growth traits at birth, 4, 9, and 16 months of age are shown in Supplementary Tables S2–S4.

#### 3.5.1 Association Analysis of *SSTR5* With Growth Traits

**FIGURE 3:**
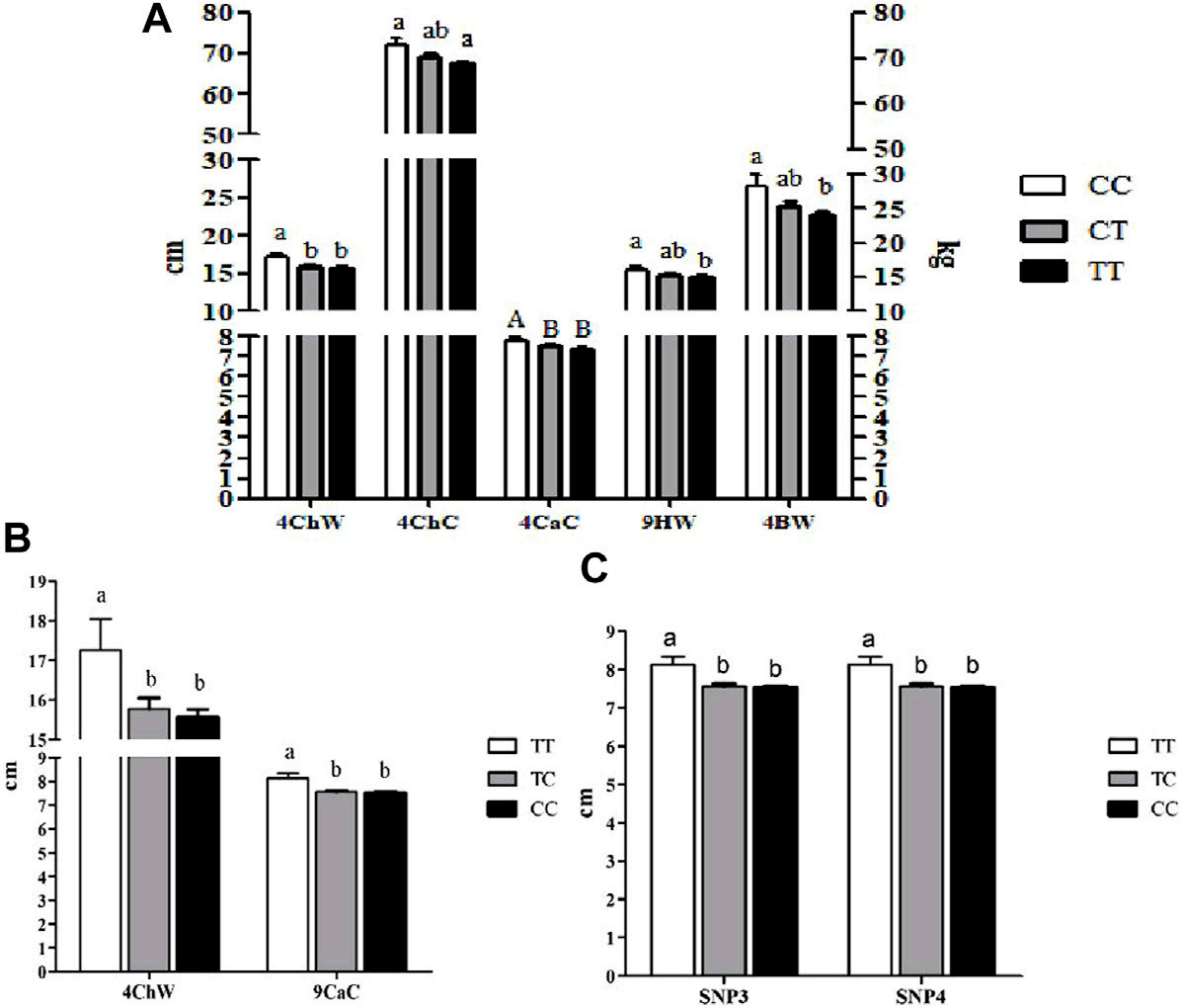
Association analyses of SNPs in *SSTR5* with growth traits of Hulun Buir sheep^1^. **(A)** Association analyses of SNP1 in *SSTR5* with growth traits of Hulun Buir sheep; 4ChW = chest width at 4 months of age; 4ChC = chest circumference at 4 months of age; 4CaC = cannon circumference at 4 months of age; 4HW = hip width at 4 months of age; 4BW = body weight at 4 months of age. **(B)** Association analyses of SNP7 in *SSTR5* with growth traits of Hulun Buir sheep; 4ChW = chest width at 4 months of age; 9CaC = cannon circumference at 9 months of age. **(C)** Association analyses of SNP3 and SNP4 in *SSTR5* with cannon circumference at 9 months of age of Hulun Buir sheep. ^a,b^ means with different superscript letters are significantly different (*p* < 0.05). ^A,B^ means with different superscript letters are very significantly different (*p* < 0.01). ^1^A group size of Population genetics analyses was *n* = 233.

The genotypes of SNP1 were significantly associated with BW, ChW, and ChC at 4 months of age and HW at 9 months of age (*p*<0.05). They also showed extremely significant association with CaC at 4 months of age (*p*<0.01). CaC at 9 months of age was significantly associated with the genotypes of SNP3, SNP4, and SNP7, and the genotypes of SNP7 were significantly associated with ChW at 4 months of age (*p*<0.05, Figure 3). No significant differences were observed among the rest of the SNPs with other growth traits (*p*<0.05).

#### 3.5.2 Haplotype Association Analysis With Growth Traits

The results of association analyses between the haplotypes and growth traits at birth, 4, 9, and 16 months of age are shown in Supplementary Table S5. There were no detectable differences among the five haplotypes of the seven SNPs in strong LD (*p*<0.05).

## 4 Discussion

This is the first report of associations between SNPs of *SSTR5* and growth traits in sheep. Growth is one of the most important economical traits monitored in domestic animals; therefore, it is a significant research topic in most genetic selection programs (Koller et al., 2020). As the main measure of growth traits, body weight and size have important impacts on the production of meat and wool (Luo, et al., 2021). In this study, we recorded these production traits from birth to adulthood in Hulun Buir sheep. We measured body weight at 4, 9, and 16 months of age, using seven body size indicators at 4 and 9 months of age, and using five body size indicators at 16 months of age. The phenotypic data contained the main growth traits of the sheep, providing a comprehensive image of growth trends in the Hulun Buir sheep. Our study was more comprehensive than most other publications and featured a longer period.

Body size indicators are important tools for accurately measuring livestock. These measurements are used to study the appearance, characteristics of the breed, and production performance. The growth rate of body size of ovine used for meat varies with age (Chao et al., 2016). In Hulun Buir sheep, we identified significant positive correlations between body size indicators and body weight, and individual size indicators affected body weight directly or indirectly. At 4 months, ChD, BL, and ChW had the strongest correlations with BW, successively. At 9 months, ChC, BH, and BL had the strongest correlation with BW, successively. At 16 months, CaC, BL, and HW had the strongest correlation with BW, successively. Moreover, with an increase in age, the correlation between body size and body weight weakened.

SNPs, defined as a substitution, insertion, or deletion of a single nucleotide, are important genetic sources for animal breeding. Gene expression and protein functions are related to the location of the SNPs in regulatory sequences or coding regions (Stevenson, 2015). Exons are protein-coding regions consisting of only 1%–2% of the genome, and the mutation rate in exons is approximately one-fifth of that in non-coding regions (Komar, 2009). However, almost 85% of reported disease-causing genes harbor mutations in their exons, which is of great significance for the study of genetic diseases (Li H et al., 2019). Thus, exome sequencing is the most efficient approach for identifying potentially functional mutations related to phenotypes in domestic animals (Li et al., 2013). Exome sequencing is also the most cost-efficient sequencing approach for conducting genome research and animal phenotyping (Guo et al., 2014), and this technology can be used to more effectively identify SNPs affecting growth traits.

Linked loci are a particular concern as there is substantial LD among causal SNPs (Koch, 2019). Studies have shown that body size is affected by the buildup of interpopulation LD among loci, caused by selection (Routtu et al., 2014). In this study, we observed LD among SNP1–7 and identified five common haplotypes in this LD region. However, there was no correlation between these haplotypes and growth traits possibly because of the small sample size or because of interactions between other genes and the SNPs in *SSTR5*.

SSTR5 reduces the activity of MAPK, which is considered a key molecule in the transduction of growth factor proliferation signals (Cattaneo et al., 1996; Cordelier et al., 1997). Thus, we speculated that SSTR5 plays an important role in reducing GH secretion (Franck et al., 2017). In addition, because these SSTR5-specific agonists are 1,000 times more powerful than other SSTR5-specific agonists in inhibiting GHRH-stimulated GH release in the primary culture of rat pituitary cells, SSTR5 is considered one of the main mediators of SST-induced inhibition of GH release (Shimon et al., 1997). Finally, *SSTR5* is involved in the regulation of ACTH release and may mediate SST-induced inhibition of insulin expression/secretion and cell proliferation by down-regulating the duodenal homolog box-1 (PDX-1) (Park et al., 2003; Zhou et al., 2012); both ACTH and PDX-1 are key hormones related to animal growth traits. Therefore, *SSTR5* may regulate the growth traits of Hulun Buir sheep. However, studies on *SSTR5* have mainly focused on tumors; there has been no prior study on the association between *SSTR5* and growth traits of livestock.

To discover the potential functional mutation related to the growth traits of Hulun Buir sheep, we conducted exome sequencing of *SSTR5* and then performed association analyses of the phenotypic records. Seven SNPs (C186T, C351T, T384C, T537C, C576T, G768A, and T989C) were all in Hardy–Weinberg equilibrium, which indicated that the population size under random mating conditions (without selection) was adequate for the experiment (Liu et al., 2018).

Synonymous mutations can affect translation dynamics and protein folding, leading to phenotypic changes (McCarthy et al., 2017; Chu and Wei, 2019). This was consistent with our findings, while SNP1, SNP3, and SNP4 did not cause synonymous mutations in amino acid sequences, SNP1 genotypes were significantly associated with BW at 4 months of age and with ChC, Chw, CaC, and HW at 9 months of age; SNP3 and SNP4 were significantly associated with CaC at 4 months of age.

These types of mutations are often deleterious (Saavedra-Rodriguez et al., 2021). In this study, SNP7 was identified as a non-synonymous mutation significantly associated with CaC at 9 months of age. The CaC of the sheep with a wild-type genotype (TT) was significantly larger than that of the sheep with two mutants (TC and CC). These results were also observed in other association analyses of SNPs and growth traits, suggesting that the SNPs identified in this study may very likely be deleterious. These findings indicate that Hulun Buir sheep may exhibit a tendency for breed deterioration; therefore, further studies on the effect of *SSTR5* on the growth traits of Hulun Buir sheep are necessary.

Analyses of the physicochemical properties of SNP7 indicated that the wild-type SSTR5 protein had two O-glycosylation sites, whereas the mutant had only one. The O-glycosylated stalk domain serves as a functional element for delivering proteins to the apical plasma membrane (Yeaman et al., 1997) and plays diverse, highly specific roles in fine-tuning protein functions (Schjoldager and Clausen, 2012). Therefore, SNP7 mutation may affect the function of the SSTR5 protein.

Association analyses of SNPs and growth traits in different ages revealed that the genotypes of SNP1, SNP3, SNP4, and SNP7 were associated with various growth indexes at 4 and 9 months of age. We inferred that the *SSTR5* might affect the early growth and development of Hulun Buir sheep. These four SNPs were significantly correlated with CaC and may serve as molecular markers to determine CaC.

## 5 Conclusion

In the current study, we used exon sequencing technology to screen *SSTR5* and discovered seven SNPs in Hulun Buir sheep. SNP1, SNP3, SNP4, and SNP7 were associated with CaC, demonstrating potential as molecular markers for the selection of CaC in Hulun Buir sheep. The genotypes of SNP1 were also associated with BW and ChC at 4 months of age, and HW at 9 months of age, indicating that SNP1 could be used as molecular markers for the selection of growth traits in Hulun Buir sheep. These molecular markers may provide a theoretical basis for improving the growth traits of Hulun Buir sheep.

## Data Availability

The datasets presented in this study can be found in online repositories. The names of the repository/repositories and accession number(s) can be found in the article/Supplementary Material.

## References

[B1] Al-Mamun H. A. Kwan P. Clark S. A. Ferdosi M. H. Tellam R. Gondro C. (2015). Genome-wide Association Study of Body Weight in Australian Merino Sheep Reveals an Orthologous Region on OAR6 to Human and Bovine Genomic Regions Affecting Height and Weight. Genet. Sel Evol. 47 (1), 66. 10.1186/s12711-015-0142-4 26272623 PMC4536601

[B2] Anzola L. K. Rivera J. N. Dierckx R. A. Lauri C. Valabrega S. Galli F. (2019). Value of Somatostatin Receptor Scintigraphy with 99mTc-HYNIC-TOC in Patients with Primary Sjögren Syndrome. Jcm 8 (6), 763. 10.3390/jcm8060763 PMC6616389 31151155

[B3] Barrett J. C. Fry B. Maller J. Daly M. J. (2005). Haploview: Analysis and Visualization of LD and Haplotype Maps. Bioinformatics 21 (2), 263–265. 10.1093/bioinformatics/bth457 15297300

[B4] Brazeau P. Vale W. Burgus R. Ling N. Butcher M. Rivier J. (1973). Hypothalamic Polypeptide that Inhibits the Secretion of Immunoreactive Pituitary Growth Hormone. Science 179 (4068), 77–79. 10.1126/science.179.4068.77 4682131

[B5] Cattaneo M. G. Amoroso D. Gussoni G. Sanguini A. M. Vicentini L. M. (1996). A Somatostatin Analogue Inhibits MAP Kinase Activation and Cell Proliferation in Human Neuroblastoma and in Human Small Cell Lung Carcinoma Cell Lines. FEBS Lett. 397 (2-3), 164–168. 10.1016/s0014-5793(96)01159-3 8955339

[B6] Chao T. Wang G. Wang J. Liu Z. Ji Z. Hou L. (2016). Identification and Classification of New Transcripts in Dorper and Small-Tailed Han Sheep Skeletal Muscle Transcriptomes. PloS one 11 (7), e0159638. 10.1371/journal.pone.0159638 27434270 PMC4951087

[B7] Chu D. Wei L. (2019). Nonsynonymous, Synonymous and Nonsense Mutations in Human Cancer-Related Genes Undergo Stronger Purifying Selections Than Expectation. BMC cancer 19 (1), 359. 10.1186/s12885-019-5572-x 30991970 PMC6469204

[B8] Colturi T. J. Unger R. H. Feldman M. (1984). Role of Circulating Somatostatin in Regulation of Gastric Acid Secretion, Gastrin Release, and Islet Cell Function. Studies in Healthy Subjects and Duodenal Ulcer patientsStudies in Healthy Subjects and Duodenal Ulcer Patients. J. Clin. Invest. 74 (2), 417–423. 10.1172/jci111437 6146638 PMC370492

[B9] Cordelier P. Esteve J.-P. Bousquet C. Delesque N. O'Carroll A.-M. Schally A. V. (1997). Characterization of the Antiproliferative Signal Mediated by the Somatostatin Receptor Subtype Sst5. Proc. Natl. Acad. Sci. 94 (17), 9343–9348. 10.1073/pnas.94.17.9343 9256484 PMC23188

[B10] Cyranoski D. (2015). Super-muscly Pigs Created by Small Genetic Tweak. Nature 523 (7558), 13–14. 10.1038/523013a 26135425

[B11] Fagan S. P. Azizzadeh A. Moldovan S. Ray M. K. Adrian T. E. Ding X. (1998). Insulin Secretion Is Inhibited by Subtype Five Somatostatin Receptor in the Mouse. Surgery 124 (2), 254–259. 10.1016/S0039-6060(98)70128-X 9706146

[B12] Finley J. C. W. Maderdrut J. L. Roger L. J. Petrusz P. (1981). The Immunocytochemical Localization of Somatostatin-Containing Neurons in the Rat central Nervous System. Neuroscience 6 (11), 2173–2192. 10.1016/0306-4522(81)90006-3 6120483

[B13] Franck S. E. Gatto F. van der Lely A. J. Janssen J. A. M. J. L. Dallenga A. H. G. Nagtegaal A. P. (2017). Somatostatin Receptor Expression in GH-Secreting Pituitary Adenomas Treated with Long-Acting Somatostatin Analogues in Combination with Pegvisomant. Neuroendocrinology 105 (1), 44–53. 10.1159/000448429 27455094 PMC5475231

[B14] Guo Y. Zhao S. Lehmann B. D. Sheng Q. Shaver T. M. Stricker T. P. (2014). Detection of Internal Exon Deletion with Exon Del. BMC bioinformatics 15 (1), 332. 10.1186/1471-2105-15-332 25322818 PMC4288651

[B15] Keller T. E. Mis S. D. Jia K. E. Wilke C. O. (2012). Reduced mRNA Secondary-Structure Stability Near the Start Codon Indicates Functional Genes in Prokaryotes. Genome Biol. Evol. 4 (2), 80–88. 10.1093/gbe/evr12910.1093/gbe/evr129 22138151 PMC3269970

[B16] Koch E. M. (2019). The Effects of Demography and Genetics on the Neutral Distribution of Quantitative Traits. Genetics 211 (4), 1371–1394. 10.1534/genetics.118.301839 30782599 PMC6456309

[B17] Koller D. Saiz‐Rodríguez M. Zubiaur P. Ochoa D. Almenara S. Román M. (2020). The Effects of Aripiprazole and Olanzapine on Pupillary Light Reflex and its Relationship with Pharmacogenetics in a Randomized Multiple‐dose Trial. Br. J. Clin. Pharmacol. 86 (10), 2051–2062. 10.1111/bcp.14300 32250470 PMC7495280

[B18] Komar A. (2009). Single Nucleotide Polymorphisms : Methods and Protocols. Humana Press. 10.1007/978-1-60327-411-1 Single Nucleotide Polymorphisms

[B19] Koressaar T. Remm M. (2007). Enhancements and Modifications of Primer Design Program Primer3. Bioinformatics 23 (10), 1289–1291. 10.1093/bioinformatics/btm091 17379693

[B20] La Y. Zhang X. Li F. Zhang D. Li C. Mo F. (2019). Molecular Characterization and Expression of SPP1, LAP3 and LCORL and Their Association with Growth Traits in Sheep. Genes 10 (8), 616. 10.3390/genes10080616 PMC6723280 31416156

[B21] Li C. Li M. Li X. Ni W. Xu Y. Yao R. (2019). Whole-Genome Resequencing Reveals Loci Associated with Thoracic Vertebrae Number in Sheep. Front. Genet. 10, 674. 10.3389/fgene.2019.00674 31379930 PMC6657399

[B22] Li H. Yang H. Lv N. Ma C. Li J. Shang Q. (2019). Whole Exome Sequencing and Methylation-specific M-ultiplex L-igation-dependent P-robe A-mplification A-pplied to I-dentify Angelman S-yndrome D-ue to P-aternal U-niparental D-isomy in T-wo U-nrelated P-atients. Mol. Med. Rep. 20 (2), 1178–1186. 10.3892/mmr.2019.10339 31173236 PMC6625451

[B23] Li X. Luo R. Mo X. Jiang R. Kong H. Hua W. (2013). Polymorphism of ZBTB17 Gene Is Associated with Idiopathic Dilated Cardiomyopathy: a Case Control Study in a Han Chinese Population. Eur. J. Med. Res. 18 (1), 10. 10.1186/2047-783X-18-10 23570452 PMC3626695

[B24] Liu B. An T. Li M. Yi Z. Li C. Sun X. (2018). The Association between Early-Onset Cardiac Events Caused by Neoadjuvant or Adjuvant Chemotherapy in Triple-Negative Breast Cancer Patients and Some Novel Autophagy-Related Polymorphisms in Their Genomic DNA: a Real-World Study. Cancer Commun. 38 (1), 71. 10.1186/s40880-018-0343-7 PMC6280434 30514381

[B25] Lloyd K. C. Amirmoazzami S. Friedik F. Chew P. Walsh J. H. (1997). Somatostatin Inhibits Gastrin Release and Acid Secretion by Activating Sst2 in Dogs. Am. J. Physiol. 272 (6 Pt 1), G1481–G1488. 10.1152/ajpgi.1997.272.6.G1481 9227485

[B26] Luo W. Zhou Y. Wang J. Yu X. Tong J. (2021). Identifying Candidate Genes Involved in the Regulation of Early Growth Using Full-Length Transcriptome and RNA-Seq Analyses of Frontal and Parietal Bones and Vertebral Bones in Bighead Carp (Hypophthalmichthys Nobilis). Front. Genet. 11, 603454. 10.3389/fgene.2020.603454 33519908 PMC7844397

[B27] Luque R. M. Gahete M. D. Hochgeschwender U. Kineman R. D. (2006). Evidence that Endogenous SST Inhibits ACTH and Ghrelin Expression by Independent Pathways. Am. J. Physiology-Endocrinology MetabolismEndocrinology Metab. 291 (2), E395–E403. 10.1152/ajpendo.00038.2006 16825606

[B28] Maecke H. R. Reubi J. C. (2011). Somatostatin Receptors as Targets for Nuclear Medicine Imaging and Radionuclide Treatment. J. Nucl. Med. 52 (6), 841–844. 10.2967/jnumed.110.084236 21571797

[B29] McCarthy C. Carrea A. Diambra L. (2017). Bicodon Bias Can Determine the Role of Synonymous SNPs in Human Diseases. BMC genomics 18 (1), 227. 10.1186/s12864-017-3609-6 28288557 PMC5347174

[B30] Melmed S. (2003). Mechanisms for Pituitary Tumorigenesis: the Plastic Pituitary. J. Clin. Invest. 112 (11), 1603–1618. 10.1172/jci20401 14660734 PMC281651

[B31] Nei M. Roychoudhury A. K. (1974). Sampling Variances of Heterozygosity and Genetic Distance. Genetics 76 (2), 379–390. 10.1093/genetics/76.2.379 4822472 PMC1213072

[B32] Ortega M. S. Denicol A. C. Cole J. B. Null D. J. Hansen P. J. (2016). Use of Single Nucleotide Polymorphisms in Candidate Genes Associated with Daughter Pregnancy Rate for Prediction of Genetic merit for Reproduction in Holstein Cows. Anim. Genet. 47 (3), 288–297. 10.1111/age.12420 26923315

[B33] Park S. Kamegai J. Kineman R. D. (2003). Role of Glucocorticoids in the Regulation of Pituitary Somatostatin Receptor Subtype (Sst1-sst5) mRNA Levels: Evidence for Direct and Somatostatin-Mediated Effects. Neuroendocrinology 78 (3), 163–175. 10.1159/000072798 14512709

[B34] Quan F. B. Desban L. Mirat O. Kermarquer M. Roussel J. Koëth F. (2020). Somatostatin 1.1 Contributes to the Innate Exploration of Zebrafish Larva. Sci. Rep. 10 (1), 15235. 10.1038/s41598-020-72039-x 32943676 PMC7499426

[B35] Routtu J. Hall M. D. Albere B. Beisel C. Bergeron R. D. Chaturvedi A. (2014). An SNP-Based Second-Generation Genetic Map of Daphnia magna and its Application to QTL Analysis of Phenotypic Traits. BMC genomics 15 (1), 1033. 10.1186/1471-2164-15-1033 25431334 PMC4301878

[B36] Saavedra-Rodriguez K. Campbell C. L. Lozano S. Penilla-Navarro P. Lopez-Solis A. Solis-Santoyo F. (2021). Permethrin Resistance in *Aedes aegypti*: Genomic Variants that Confer Knockdown Resistance, Recovery, and Death. Plos Genet. 17 (6), e1009606. 10.1371/journal.pgen.1009606 34138859 PMC8211209

[B37] Schjoldager K. T.-B. G. Clausen H. (2012). Site-specific Protein O-Glycosylation Modulates Proprotein Processing - Deciphering Specific Functions of the Large Polypeptide GalNAc-Transferase Gene Family. Biochim. Biophys. Acta (Bba) - Gen. Subjects 1820, 2079–2094. 10.1016/j.bbagen.2012.09.014 23022508

[B38] Shimon I. Taylor J. E. Dong J. Z. Bitonte R. A. Kim S. Morgan B. (1997). Somatostatin Receptor Subtype Specificity in Human Fetal Pituitary Cultures. Differential Role of SSTR2 and SSTR5 for Growth Hormone, Thyroid-Stimulating Hormone, and Prolactin Regulation. J. Clin. Invest. 99 (4), 789–798. 10.1172/JCI119225 9045884 PMC507864

[B39] Stevenson K. (2015). Genetic Diversity of *Mycobacterium avium* Subspecies Paratuberculosis and the Influence of Strain Type on Infection and Pathogenesis: a Review. Vet. Res. 46 (1), 64. 10.1186/s13567-015-0203-2 26092160 PMC4473831

[B40] Strowski M. Z. Parmar R. M. Blake A. D. Schaeffer J. M. (2000). Somatostatin Inhibits Insulin and Glucagon Secretion via Two Receptor Subtypes: An *In Vitro* Study of Pancreatic Islets from Somatostatin Receptor 2 Knockout Mice*. Endocrinology 141 (1), 111–117. 10.1210/endo.141.1.7263 10614629

[B41] Tulassay Z. (1998). Somatostatin and the Gastrointestinal Tract. Scand. J. Gastroenterol. 33, 115–121. Supplement. 10.1080/003655298750026642 9867121

[B42] Wang H. Zhang L. Cao J. Wu M. Ma X. Liu Z. (2015). Genome-Wide Specific Selection in Three Domestic Sheep Breeds. PloS one 10 (6), e0128688. 10.1371/journal.pone.0128688 26083354 PMC4471085

[B43] Yeaman C. Gall A. H. L. Baldwin A. N. Monlauzeur L. Bivic A. L. Rodriguez-Boulan E. (1997). The O-Glycosylated Stalk Domain Is Required for Apical Sorting of Neurotrophin Receptors in Polarized MDCK Cells. J. Cel. Biol. 139 (4), 929–940. 10.1083/jcb.139.4.929 PMC2139957 9362511

[B44] Zhang L. N. Wu P. Xuan C. Z. Liu Y. Q. Wu J. (2016). Advance in Body Size Measurement and Conformation Appraisal for Sheep. Trans. Chin. Soc. Agric. Eng. 32 (S1), 190–197.

[B45] Zhou G. Liu S.-H. Shahi K. M. Wang H. Duan X. Lin X. (2012). Negative Regulation of Pancreatic and Duodenal Homeobox-1 by Somatostatin Receptor Subtype 5. Mol. Endocrinol. (Baltimore, Md 26 (7), 1225–1234. 10.1210/me.2012-1095 PMC3385795 22669743

