# Supplementary material for: Identification of SSTR5 Gene Polymorphisms and Their Association With Growth Traits in Hulun Buir Sheep
Source: Front Genet. 2022 Apr 26;13:831599. doi: 10.3389/fgene.2022.831599 (PMC9086292; doi:10.3389/fgene.2022.831599)
Supplement: Supplementary file 1 [file Table1.DOCX]

**Supplementary Table S1.** Comparison of growth traits of extreme populations

| Traits | Slow-growing | Fast-growing |  | *P-value* |
| --- | --- | --- | --- | --- |
| BRW/kg | 3.86±0.06^B^ | 4.53±0.05^a^ | 4.18±0.02^b^ | 0.012 |
| 4BW/kg | 17.24±0.31^C^ | 29.31±0.34^A^ | 23.96±0.17^B^ | 0.000 |
| 4BL/cm | 51.82±0.34^C^ | 60.03±0.30^A^ | 56.26±0.15^B^ | 0.000 |
| 4BH/cm | 52.49±0.55^C^ | 61.81±0.39^A^ | 57.92±0.17^B^ | 0.000 |
| 4ChW/cm | 14.37±1.60^C^ | 16.69±0.18^A^ | 15.96±0.19^B^ | 0.000 |
| 4ChD/cm | 26.08±0.21^C^ | 30.08±0.20^A^ | 28.26±0.09^B^ | 0.000 |
| 4ChC/cm | 64.35±0.58^B^ | 72.40±0.57^A^ | 69.41±0.19^C^ | 0.000 |
| 4HW/cm | 11.61±0.12^B^ | 13.26±0.11^A^ | 12.71±0.05^A^ | 0.000 |
| 4CaC/cm | 7.07±0.04^C^ | 7.85±0.04^A^ | 7.55±0.02^B^ | 0.000 |
| 9BW/kg | 25.14±0.22^C^ | 39.49±0.18^A^ | 32.84±0.17^B^ | 0.000 |
| 9BL/cm | 60.65±0.26^C^ | 66.73±0.32^A^ | 62.26±0.15^B^ | 0.000 |
| 9BH/cm | 62.63±0.25^C^ | 70.80±0.31^A^ | 66.91±0.17^B^ | 0.000 |
| 9ChC/cm | 76.99±0.42^C^ | 89.88±0.42^A^ | 80.41±0.19^B^ | 0.000 |
| 9ChD/cm | 32.82±0.21^B^ | 34.50±0.23^A^ | 33.42±0.21B | 0.000 |
| 9ChW/cm | 20.07±0.18^C^ | 23.25±0.21^A^ | 21.69±0.11^B^ | 0.000 |
| 9HW/cm | 13.53±0.29^B^ | 15.77±0.12^A^ | 14.58±0.32^A^ | 0.000 |
| 9CaC/cm | 7.18±0.04^C^ | 7.96±0.05^A^ | 7.55±0.02^B^ | 0.000 |
| 16BW/kg | 34.67±0.51^C^ | 41.86±0.50^A^ | 38.98±0.15^B^ | 0.000 |
| 16BL/cm | 65.27±0.38^B^ | 69.40±0.46^A^ | 67.35±0.12^B^ | 0.000 |
| 16BH/cm | 69.80±0.84^C^ | 75.23±0.61^A^ | 72.04±0.20^B^ | 0.000 |
| 16ChD/cm | 33.05±0.57^C^ | 34.82±0.20^A^ | 34.05±0.08^B^ | 0.000 |
| 16HW/cm | 17.34±0.19^B^ | 18.73±0.19^A^ | 18.52±0.05^A^ | 0.000 |
| 16CaC/cm | 8.16±0.10 | 8.28±0.04 | 8.20±0.01 | 0.286 |
| Growth rate (0-16) | 0.05±0.00^C^ | 0.07±0.01^A^ | 0.06±0.01^B^ | 0.000 |

BRW = birth weight at 4 months of age; 4BW = body weight at 4 months of age; 4BL = body length at 4 months of age; 4BH = body height at 4 months of age; 4ChC = chest circumference at 4 months of age; 4ChD = chest depth at 4 months of age; 4ChW = chest width at 4 months of age, 4HW = hip width at 4 months of age; 4CaC = cannon circumference at 4 months of age.9BW = body weight at 9 months of age; 9BL = body length at 9 months of age; 9BH = body height at 9 months of age; 9ChC = chest circumference at 9 months of age; 9ChD = chest depth at 9 months of age; 9ChW = chest width at 9 months of age, 9HW = hip width at 9 months of age; 9CaC = cannon circumference at 9 months of age. 16BW = body weight at 16 months of age; 16BL = body length at 16 months of age; 16BH = body height at 16 months of age; 16ChC = chest circumference at 16 months of age; 16ChD = chest depth at 16 months of age; 16ChW = chest width at 16 months of age; 16HW = hip widthat 16 months of age; 16CaC = cannon circumference at 16 months of age.

^1^Data represent means ± SEM (n = 233).
