# Supplementary material for: Identification of SSTR5 Gene Polymorphisms and Their Association With Growth Traits in Hulun Buir Sheep
Source: Front Genet. 2022 Apr 26;13:831599. doi: 10.3389/fgene.2022.831599 (PMC9086292; doi:10.3389/fgene.2022.831599)
Supplement: Supplementary file 2 [file Table2.DOCX]

**Supplementary Table S2.** Association analyses of SNPs and genotypes in *SSTR5* with growth traits of Hulun Buir sheep at birth and 4 months of age^1^

| SNP | Genotype frequency | BRW/kg | BW/kg | BL/cm | BH/cm | ChW/cm | ChD/cm | ChC/cm | HW/cm | CaC/cm |
| --- | --- | --- | --- | --- | --- | --- | --- | --- | --- | --- |
| SNP1 | CC (*n* = 18) | 4.25±0.16 | 26.48±1.64^a^ | 58.06±1.27 | 58.78±1.63 | 17.10±0.53^a^ | 28.51±0.71 | 72.07±1.72^a^ | 12.96±0.35 | 7.79±0.14^A^ |
|  | CT (*n* = 93) | 4.26±0.07 | 23.85±0.72^ab^ | 56.09±0.56 | 57.55±0.72 | 15.83±0.23^b^ | 28.16±0.31 | 69.23±0.76^ab^ | 12.54±0.15 | 7.54±0.06^B^ |
|  | TT (*n* = 122) | 4.16±0.06 | 22.61±0.63^b^ | 55.66±0.49 | 56.70±0.63 | 15.56±0.21^b^ | 28.02±0.27 | 67.32±0.66^b^ | 12.32±0.13 | 7.38±0.05^B^ |
| SNP2 | CC (*n* = 89) | 4.24±0.07 | 23.83±0.75 | 56.49±0.63 | 57.20±0.82 | 15.94±0.26 | 27.83±0.35 | 68.50±0.85 | 12.62±0.17 | 7.47±0.07 |
|  | CT (*n* = 122) | 4.27±0.08 | 23.27±0.66 | 55.67±0.58 | 57.15±0.75 | 15.62±0.24 | 28.29±0.32 | 68.13±0.77 | 12.41±0.15 | 7.48±0.06 |
|  | TT (*n* = 32) | 4.15±0.12 | 22.71±1.25 | 55.27±1.23 | 56.79±1.48 | 15.32±0.47 | 28.00±0.62 | 67.66±1.52 | 12.40±0.30 | 7.39±0.12 |
| SNP3 | TT (*n* = 9) | 4.15±0.24 | 26.01±2.50 | 55.94±1.95 | 57.81±2.55 | 17.25±0.80 | 27.63±1.08 | 72.81±2.59 | 13.13±0.51 | 7.75±0.20 |
|  | TC (*n* = 79) | 4.29±0.08 | 23.55±0.87 | 55.92±0.68 | 57.41±0.89 | 15.76±0.28 | 28.21±0.38 | 68.74±0.90 | 12.64±0.18 | 7.55±0.07 |
|  | CC (*n* = 145) | 4.21±0.06 | 22.81±0.65 | 55.90±0.51 | 56.92±0.66 | 15.58±0.21 | 28.02±0.28 | 67.62±0.67 | 12.37±0.13 | 7.39±0.05 |
| SNP4 | TT (*n* = 9) | 4.15±024 | 26.01±2.50 | 56.94±1.95 | 57.81±2.22 | 17.25±0.80 | 27.63±1.08 | 72.81±2.60 | 13.13±0.51 | 7.75±0.20 |
|  | TC (*n* = 82) | 4.29±0.08 | 23.53±0.86 | 55.91±0.68 | 57.39±0.88 | 15.76±0.28 | 28.18±0.37 | 68.65±0.90 | 12.64±0.18 | 7.55±0.07 |
|  | CC (*n* = 142) | 4.21±0.06 | 22.82±0.65 | 55.90±0.51 | 56.93±0.66 | 15.57±0.21 | 28.03±0.28 | 67.66±0.68 | 12.36±0.13 | 7.39±0.05 |
| SNP5 | CC (*n* = 18) | 4.19±0.18 | 25.60±1.83 | 57.70±1.42 | 58.26±1.86 | 16.79±0.59 | 27.82±0.79 | 71.36±1.90 | 12.89±0.38 | 7.69±0.15 |
|  | CT (*n* = 93) | 4.27±0.07 | 23.39±0.80 | 55.89±0.62 | 57.32±0.81 | 15.67±0.26 | 28.14±0.34 | 68.62±0.83 | 12.52±0.16 | 7.52±0.06 |
|  | TT (*n* = 122) | 4.21±0.06 | 22.68±0.71 | 55.73±0.55 | 56.79±0.72 | 15.57±0.23 | 28.05±0.31 | 67.42±0.74 | 12.41±0.15 | 7.38±0.06 |
| SNP6 | GG (*n* = 105) | 4.25±0.07 | 23.67±0.76 | 56.54±0.59 | 57.30±0.77 | 15.96±0.24 | 27.95±0.33 | 68.61±0.79 | 12.60±0.16 | 7.50±0.06 |
|  | GA (*n* = 114) | 4.20±0.07 | 22.78±0.73 | 55.59±0.57 | 57.06±0.74 | 15.48±0.24 | 28.27±0.32 | 67.83±0.77 | 12.36±0.15 | 7.44±0.06 |
|  | AA (*n* = 14) | 4.37±0.21 | 22.89±2.14 | 54.32±1.66 | 56.24±2.18 | 15.60±0.69 | 27.29±0.92 | 68.43±2.24 | 12.74±0.44 | 7.34±0.17 |
| SNP7 | TT (*n* = 9) | 4.15±0.24 | 26.01±2.50 | 56.94±1.95 | 57.81±2.55 | 17.25±0.80^a^ | 27.63±1.08 | 72.81±2.59 | 13.13±0.51 | 7.75±0.20 |
|  | TC (*n* = 83) | 4.30±0.08 | 23.67±0.86 | 56.00±0.67 | 57.45±0.87 | 15.78±0.27^b^ | 28.21±0.37 | 68.74±0.89 | 12.65±0.18 | 7.56±0.07 |
|  | CC (*n* = 141) | 4.20±0.06 | 22.73±0.65 | 55.85±0.51 | 56.89±0.67 | 15.56±0.21^b^ | 28.02±0.28 | 67.60±0.68 | 12.35±0.13 | 7.39±0.05 |

BRW = birth weight; BW = body weigh; BL = body length; BH = body height; ChC = chest circumference; ChD = chest depth; ChW = chest width; HW = hip width; CaC = cannon circumference.

^a,b^ Within a row, means with different superscript letters are significantly different (*P* < 0.05).

^A,B^ Within a row, means with different superscript letters are very significantly different (*P* < 0.01).

^1^Data represent means ± SEM (*n* = 233).
