# Supplementary material for: Identification of SSTR5 Gene Polymorphisms and Their Association With Growth Traits in Hulun Buir Sheep
Source: Front Genet. 2022 Apr 26;13:831599. doi: 10.3389/fgene.2022.831599 (PMC9086292; doi:10.3389/fgene.2022.831599)
Supplement: Supplementary file 3 [file Table5.DOCX]

**Supplementary Table S5.** Association analyses of haplotypes in *SSTR5* with growth traits of Hulun Buir sheep^1^

| Traits | TCCCTGC  (n = 159) | TTCCTAC  (n = 84) | CCTTCGT  (n = 33) | TTCCTGC  (n = 96) | CCCCCGC  (n = 89) |
| --- | --- | --- | --- | --- | --- |
| BRW/kg | 4.24±0.50 | 4.21±0.07 | 4.41±0.11 | 4.23±0.07 | 4.27±0.07 |
| 4BW/kg | 23.00±0.57 | 22.75±0.78 | 24.40±1.24 | 23.11±0.73 | 23.52±0.76 |
| 4BL/cm | 55.88±0.44 | 55.51±0.61 | 56.22±0.97 | 55.67±0.57 | 56.11±0.59 |
| 4BH/cm | 57.07±0.61 | 57.07±0.84 | 58.08±1.34 | 57.17±0.79 | 57.41±0.82 |
| 4ChW/cm | 15.66±0.17 | 15.54±0.24 | 16.02±0.38 | 15.61±0.22 | 15.72±0.23 |
| 4ChD/cm | 28.10±0.25 | 28.23±0.35 | 28.45±0.55 | 28.29±0.32 | 28.12±0.33 |
| 4ChC/cm | 68.00±0.59 | 67.97±0.82 | 69.64±1.30 | 68.13±0.76 | 68.70±0.79 |
| 4HW/cm | 12.46±0.11 | 12.40±0.15 | 12.70±0.24 | 12.41±0.14 | 12.53±0.15 |
| 4CaC/cm | 7.45±0.05 | 7.44±0.06 | 7.67±0.10 | 7.48±0.06 | 7.53±0.06 |
| 9BW/kg | 31.91±0.60 | 32.04±0.83 | 33.72±1.33 | 32.29±0.78 | 32.23±0.81 |
| 9BL/cm | 63.74±0.35 | 63.53±0.48 | 64.25±0.77 | 63.61±0.45 | 63.55±0.47 |
| 9BH/cm | 66.60±0.41 | 66.41±0.56 | 67.64±0.90 | 66.50±0.53 | 66.77±0.55 |
| 9ChC/cm | 82.88±0.64 | 83.17±0.88 | 84.73±1.41 | 83.27±0.83 | 83.17±0.86 |
| 9ChD/cm | 32.45±0.24 | 32.63±0.33 | 32.92±0.53 | 32.59±0.31 | 32.48±0.32 |
| 9ChW/cm | 21.61±0.21 | 21.79±0.29 | 21.66±0.47 | 21.79±0.28 | 21.24±0.29 |
| 9HW/cm | 14.48±0.12 | 14.54±0.17 | 14.84±0.26 | 14.55±0.16 | 14.64±0.16 |
| 9CaC/cm | 7.53±0.05 | 7.54±0.07 | 7.68±0.11 | 7.58±0.07 | 7.55±0.07 |
| 16BW/kg | 38.20±0.47 | 37.40±0.65 | 38.19±1.04 | 37.94±0.61 | 38.42±0.63 |
| 16BL/cm | 67.31±0.39 | 66.94±0.53 | 67.19±0.85 | 67.15±0.50 | 67.49±0.52 |
| 16BH/cm | 72.15±0.61 | 72.11±0.84 | 72.66±1.34 | 72.19±0.79 | 72.35±0.82 |
| 16ChD/cm | 34.11±0.27 | 33.85±0.37 | 33.73±0.59 | 33.84±0.34 | 33.92±0.36 |
| 16HW/cm | 17.99±0.16 | 17.98±0.21 | 17.50±0.34 | 17.90±0.20 | 18.11±0.21 |
| 16CaC/cm | 8.22±0.05 | 8.12±0.06 | 8.14±0.10 | 8.13±0.06 | 8.17±0.06 |

BRW = birth weight at 4 months of age; 4BW = body weight at 4 months of age; 4BL = body length at 4 months of age; 4BH = body height at 4 months of age; 4ChC = chest circumference at 4 months of age; 4ChD = chest depth at 4 months of age; 4ChW = chest width at 4 months of age, 4HW = hip width at 4 months of age; 4CaC = cannon circumference at 4 months of age.9BW = body weight at 9 months of age; 9BL = body length at 9 months of age; 9BH = body height at 9 months of age; 9ChC = chest circumference at 9 months of age; 9ChD = chest depth at 9 months of age; 9ChW = chest width at 9 months of age, 9HW = hip width at 9 months of age; 9CaC = cannon circumference at 9 months of age. 16BW = body weight at 16 months of age; 16BL = body length at 16 months of age; 16BH = body height at 16 months of age; 16ChC = chest circumference at 16 months of age; 16ChD = chest depth at 16 months of age; 16ChW = chest width at 16 months of age; 16HW = hip widthat 16 months of age; 16CaC = cannon circumference at 16 months of age.

^1^Data represent means ± SEM (n = 233).
