# Supplementary material for: Identification of SSTR5 Gene Polymorphisms and Their Association With Growth Traits in Hulun Buir Sheep
Source: Front Genet. 2022 Apr 26;13:831599. doi: 10.3389/fgene.2022.831599 (PMC9086292; doi:10.3389/fgene.2022.831599)
Supplement: Supplementary file 4 [file Table3.DOCX]

**Supplementary Table S3.** Association analyses of SNPs and genotypes in *SSTR5* with growth traits of Hulun Buir sheep at 9 months of age^1^

| SNP | Genotype frequency | BW/kg | BL/cm | BH/cm | ChW/cm | ChD/cm | ChC/cm | HW/cm | CaC/cm |
| --- | --- | --- | --- | --- | --- | --- | --- | --- | --- |
| SNP1 | CC (*n* = 18) | 35.42±1.82 | 64.44±1.06 | 68.40±1.27 | 21.53±0.64 | 34.08±0.74 | 84.08±1.95 | 15.35±0.39^a^ | 7.86±0.15 |
|  | CT (*n* = 93) | 32.64±0.78 | 63.71±0.44 | 66.98±0.53 | 21.41±0.27 | 32.51±0.31 | 83.79±0.82 | 14.74±0.16^ab^ | 7.57±0.06 |
|  | TT (*n* = 122) | 31.54±0.69 | 63.63±0.39 | 66.18±0.47 | 21.81±0.24 | 32.54±0.27 | 82.75±0.72 | 14.4b7±0.14^b^ | 7.53±0.06 |
| SNP2 | CC (*n* = 89) | 31.98±0.86 | 63.98±0.49 | 67.06±0.58 | 21.40±0.30 | 32.59±0.35 | 82.60±0.89 | 14.54±0.17 | 7.54±0.07 |
|  | CT (*n* = 122) | 32.28±0.79 | 63.63±0.45 | 66.49±0.53 | 21.78±0.27 | 32.59±0.32 | 83.24±0.82 | 14.55±0.16 | 7.58±0.06 |
|  | TT (*n* = 32) | 31.40±1.55 | 63.24±0.88 | 65.73±1.03 | 21.36±0.54 | 32.96±0.63 | 83.18±1.60 | 14.36±0.31 | 7.54±0.13 |
| SNP3 | TT (*n* = 9) | 35.40±2.66 | 65.88±1.52 | 69.00±1.78 | 22.44±0.93 | 34.56±1.07 | 84.88±2.77 | 15.31±0.54 | 8.13±0.21^a^ |
|  | TC (*n* = 79) | 32.37±0.93 | 63.73±0.53 | 67.04±0.62 | 21.41±0.32 | 32.51±0.37 | 83.41±0.96 | 14.64±0.19 | 7.56±0.07^b^ |
|  | CC (*n* = 145) | 31.65±0.69 | 63.58±0.39 | 66.24±0.46 | 21.63±0.24 | 32.58±0.28 | 82.62±0.72 | 14.40±0.14 | 7.52±0.06^b^ |
| SNP4 | TT (*n* = 9) | 35.40±2.66 | 65.88±1.52 | 69.00±1.78 | 22.44±0.93 | 34.56±1.07 | 84.88±2.77 | 15.31±0.54 | 8.13±0.21^a^ |
|  | TC (*n* = 82) | 32.31±0.92 | 63.72±0.53 | 67.03±0.61 | 21.38±0.32 | 32.47±0.37 | 83.26±0.96 | 14.64±0.19 | 7.56±0.07^b^ |
|  | CC (*n* = 142) | 31.68±0.69 | 63.58±0.40 | 66.23±0.46 | 21.63±0.24 | 32.60±0.28 | 82.69±0.72 | 14.40±0.14 | 7.52±0.06^b^ |
| SNP5 | CC (*n* = 18) | 34.55±1.95 | 64.32±1.12 | 68.27±1.30 | 21.57±0.68 | 34.04±0.78 | 83.70±2.03 | 15.24±0.39 | 7.84±0.16 |
|  | CT (*n* = 93) | 32.13±0.85 | 63.61±0.49 | 66.72±0.57 | 21.31±0.29 | 32.40±0.34 | 83.28±0.88 | 14.59±0.17 | 7.56±0.07 |
|  | TT (*n* = 122) | 31.61±0.76 | 63.72±0.43 | 66.30±0.51 | 21.79±0.26 | 32.61±0.31 | 82.64±0.79 | 14.36±0.15 | 7.52±0.06 |
| SNP6 | GG (*n* = 105) | 32.25±0.81 | 64.00±0.46 | 67.08±0.54 | 21.45±0.28 | 32.55±0.33 | 82.80±0.84 | 14.55±0.16 | 7.58±0.07 |
|  | GA (*n* = 114) | 31.92±0.78 | 63.49±0.45 | 66.19±0.52 | 21.72±0.27 | 32.63±0.32 | 83.13±0.81 | 14.50±0.16 | 7.54±0.06 |
|  | AA (*n* = 14) | 31.55±2.29 | 63.51±1.30 | 66.72±1.52 | 21.37±0.79 | 33.37±0.92 | 83.15±2.37 | 14.49±0.46 | 7.58±0.19 |
| SNP7 | TT (*n* = 9) | 35.40±2.66 | 65.88±1.77 | 69.00±1.77 | 22.44±0.93 | 34.56±1.07 | 84.88±2.77 | 15.31±0.54 | 8.13±0.21^a^ |
|  | TC (*n* = 83) | 32.43±0.91 | 63.79±0.52 | 67.13±0.61 | 21.41±0.32 | 32.52±0.37 | 83.38±0.95 | 14.68±0.18 | 7.56±0.07^b^ |
|  | CC (*n* = 141) | 32.60±0.70 | 63.54±0.40 | 66.17±0.46 | 21.62±0.24 | 32.57±0.28 | 82.62±0.72 | 14.37±0.14 | 7.52±0.06^b^ |

BW = body weight; BL = body length; BH = body height; ChC = chest circumference; ChD = chest depth; ChW = chest width , HW = hip width; CaC = cannon circumference.

^a,b^ Within a row, means with different superscript letters are significantly different (*P* < 0.05).

^1^ Data represent means ± SEM (*n* = 233).
