# Supplementary material for: Identification of SSTR5 Gene Polymorphisms and Their Association With Growth Traits in Hulun Buir Sheep
Source: Front Genet. 2022 Apr 26;13:831599. doi: 10.3389/fgene.2022.831599 (PMC9086292; doi:10.3389/fgene.2022.831599)
Supplement: Supplementary file 5 [file Table4.DOCX]

**Supplementary Table S4.**Association analyses of SNPs and genotypes in *SSTR5* with growth traits of Hulun Buir sheep at 16 months of age^1^

| SNP | Genotype frequency | BW/kg | BL/cm | BH/cm | ChD/cm | HW/cm | CaC/cm |
| --- | --- | --- | --- | --- | --- | --- | --- |
| SNP1 | CC (*n* = 18) | 39.43±1.52 | 67.33±1.15 | 72.69±1.67 | 33.33±0.61 | 17.71±0.49 | 8.28±0.09 |
|  | CT (*n* = 93) | 38.29±0.68 | 67.38±0.52 | 72.74±0.75 | 34.02±0.28 | 18.10±0.22 | 8.16±0.04 |
|  | TT (*n* = 122) | 38.06±0.62 | 67.32±0.47 | 72.92±0.68 | 33.49±0.25 | 18.13±0.20 | 8.17±0.04 |
| SNP2 | CC (*n* = 89) | 38.77±0.72 | 67.46±0.55 | 73.00±0.78 | 33.54±0.29 | 18.17±0.23 | 8.19±0.04 |
|  | CT (*n* = 122) | 37.95±0.66 | 67.16±0.50 | 72.16±0.71 | 33.75±0.26 | 17.86±0.21 | 8.12±0.04 |
|  | TT (*n* = 32) | 37.13±1.29 | 67.44±0.98 | 73.33±1.39 | 33.30±0.51 | 18.62±0.41 | 8.27±0.07 |
| SNP3 | TT (*n* = 9) | 39.13±2.24 | 66.50±1.69 | 73.63±2.41 | 33.00±0.89 | 17.19±0.72 | 8.38±0.13 |
|  | TC (*n* = 79) | 38.09±0.78 | 67.29±0.59 | 72.73±0.84 | 33.70±0.31 | 18.02±0.25 | 8.17±0.04 |
|  | CC (n = 145) | 38.15±0.58 | 67.38±0.44 | 72.53±0.63 | 33.61±0.23 | 18.17±0.19 | 8.15±0.03 |
| SNP4 | TT (*n* = 9) | 39.13±2.24 | 66.50±1.69 | 73.63±2.41 | 33.00±0.89 | 17.19±0.74 | 8.38±0.13 |
|  | TC (*n* = 82) | 38.10±0.77 | 67.30±0.58 | 72.70±0.83 | 33.72±0.31 | 18.00±0.26 | 8.17±0.04 |
|  | CC (*n* = 142) | 38.15±0.58 | 67.37±0.44 | 72.54±0.63 | 33.59±0.23 | 18.12±0.19 | 0.15±0.03 |
| SNP5 | CC (*n* = 18) | 39.56±1.63 | 67.17±1.24 | 72.00±1.76 | 32.92±0.64 | 17.74±0.55 | 8.25±0.09 |
|  | CT (*n* = 93) | 38.27±0.71 | 67.45±0.54 | 72.57±0.77 | 34.00±0.28 | 18.14±0.24 | 8.15±0.04 |
|  | TT (*n* = 122) | 37.89±0.63 | 67.23±0.48 | 72.80±0.69 | 33.41±0.25 | 18.01±0.21 | 8.16±0.04 |
| SNP6 | GG (*n* = 105) | 39.16±0.67 | 67.61±0.51 | 72.98±0.72 | 33.58±0.27 | 17.97±0.22 | 8.19±0.04 |
|  | GA (*n* = 114) | 37.34±0.65 | 66.96±0.49 | 72.10±0.70 | 33.63±0.26 | 18.00±0.22 | 8.13±0.04 |
|  | AA (*n* = 14) | 37.41±1.89 | 67.86±1.44 | 74.63±2.05 | 33.74±0.76 | 18.91±0.64 | 8.25±0.11 |
| SNP7 | TT (*n* = 9) | 39.13±2.24 | 66.50±1.69 | 73.63±2.41 | 33.00±0.89 | 17.19±0.72 | 8.38±0.13 |
|  | TC (*n* = 83) | 38.26±0.77 | 67.41±0.58 | 72.82±0.83 | 33.71±0.30 | 18.04±0.25 | 8.18±0.04 |
|  | CC (*n* = 141) | 38.06±0.59 | 67.31±0.44 | 72.47±0.63 | 33.60±0.23 | 18.16±0.19 | 8.14±0.03 |

BW = body weight; BL = body length; BH = body height; ChC = chest circumference; ChD = chest depth; ChW = chest width; HW = hip widthat; CaC = cannon circumference.

^1^ Data represent means ± SEM (*n* = 233).
