# Supplementary material for: Identification of SSTR5 Gene Polymorphisms and Their Association With Growth Traits in Hulun Buir Sheep
Source: Front Genet. 2022 Apr 26;13:831599. doi: 10.3389/fgene.2022.831599 (PMC9086292; doi:10.3389/fgene.2022.831599)
Supplement: Supplementary file 6 [file Table6.DOCX]

**Respose to the Review Comments**

We would like to thank you for your careful reading, helpful comments, and constructive suggestions, which has significantly improved the presentation of our manuscript. At the same time, I am very ashamed and sorry for many major mistakes in my manuscript. The manuscript has also been double-checked, and the typos and grammar errors we found have been corrected , and changes to our manuscript were all highlighted within the documents by using red colored text. In the following section, we summarize our responses to each comment from the reviewers. We hope that our responses have well addressed all concerns from the reviewers, and revised manuscript can be accepted for publication.

1. According to the information presented by authors (see L 84-86) Ovine SSTR5 gene transcript has 4 exons and 3 introns (ENSOARG00000014478.1) located on chromosome 24 (GenBank, Gene ID: 443202), encoding 1044 bp base (rs 812728 - 813866) and 347 amino acid residues.However, according to the information, presented in gene bank, Gene ID 443202 corresponds to the SSTR1 somatostatin receptor 1 (https://www.ncbi.nlm.nih.gov/gene/?term=443202).Somatostatin receptor 5 is presented in the Gene bank under Gene ID 443210 (https://www.ncbi.nlm.nih.gov/gene/?term=443210). This gene has 3 exons and CDS of 367 amino acids (see NCBI Reference Sequence: NP_001009265.1).I tried to find the localization of primers in SSTR5 gene using NCBI Reference Sequence: NC_056077.1. It seems that the used primers amplified two overlapped fragments covering the part of last intron and exon of SSTR5 gene. This does not match to the information presented by authors.

Thank you for your suggestion. We have revised it in the manuscript (Line 30-31).

This is because NCBI and Ensembl reference genome are different, the sheep reference genome version in NCBI is Oar_RAMbouillet_v1.0 and RAMb_v2.0 (Fig.1), while the Ensembl version is Oar_V3.1. This experiment is under the Ensembl reference (Fig. 2). When the *SSTR5* gene used the (OAR_V3-1) version as the reference genome, it has 4 exons, encoding 344 amino acids (Fig. 2)（https://asia.ensembl.org/Ovis_aries/Transcript/Exons?db=core;g=ENSOARG00000014478;r=24:812728-813866;t=ENSOART00000015760）.

1. The other issue regarding materials and methods section. The groups of animals for the study were selected based on growth rate. Growth rate is the ratio of body weight gain over a certain period of time. I suppose that the values for growth rate for entire sample as well as for two compared groups should be provided.

Thank you for your suggestion. We have revised it in the manuscript (Supplementary Table S1).

1. L 24-25 The DNAs (???) were amplified using primers by PCR and sequenced by Sanger sequencing. Please, specify.

Thank you for your suggestion. We have revised it in the manuscript (Line 24-25).

1. L 30-31 The other SNPs (???) did not change the amino acid coding sequence except SNP7. Which SNPs do you mean?

Thank you for your suggestion. We have revised it in the manuscript (Line 30-31).

1. L 126-128 The SSTR5 gene transcript has four exons (ENSOARG00000014478.1), and one primer pair was designed covering exons 1 to 2 and another one between exons 3 and 4.This information does not correspond to the data presented in NCBI Gene Bank (see comments above). To clarify this issue, please, provide in the supplemental materials the schematic representation of SSTR5 gene (exon / intron structure) with indication of primer localizations and identified SNPs with indications of their positions in SSTR5 gene sequence (please, provide the accession number for the used sequence).

Thank you for your suggestion.The SNPs found in this experiment are all recorded in the Ensembl database (Figure 3), we have checked the SNPs information again and again to ensure that there is no problem. In Ensembl database with OAR_V3.1 reference genome, SSTR5 has 4 exons.


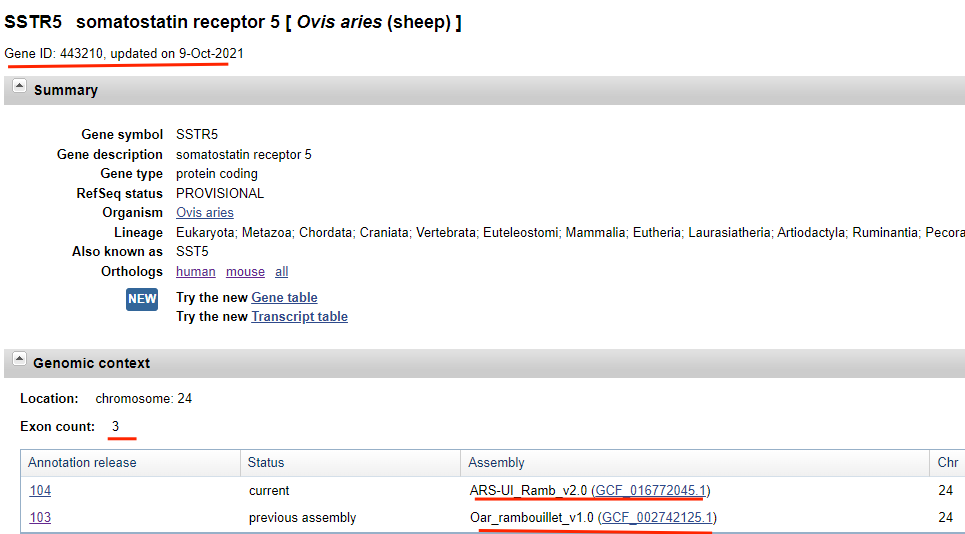
Fig 1. Information of SSTR5 gene in NCBI


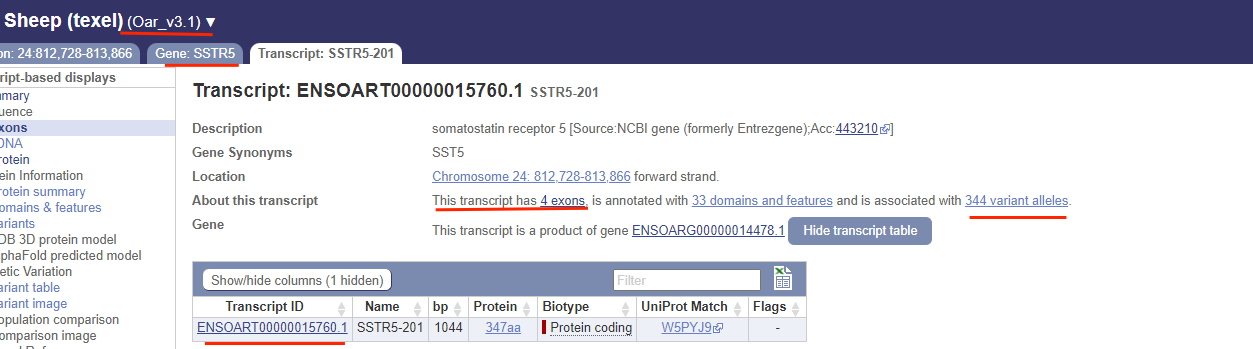
Fig 2. Information of SSTR5 gene in Ensembl


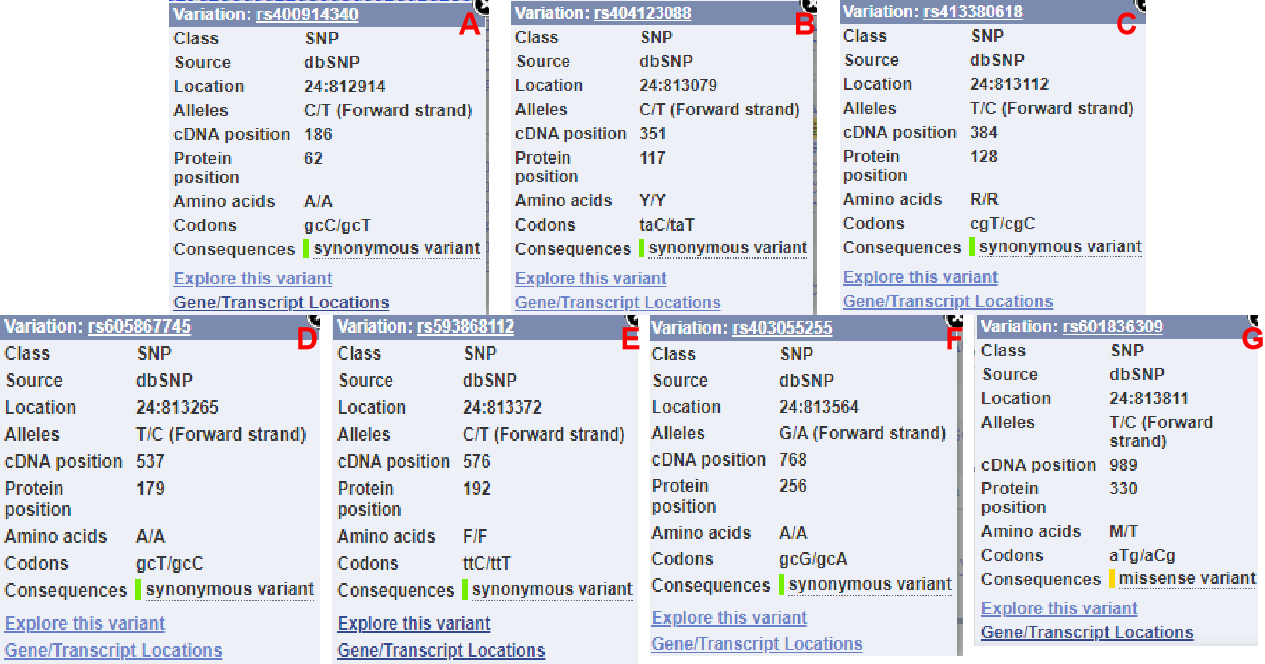


Fig 3.SNPs information of *SSTR5* gene in Ensembl

1. I strongly recommend taking into account all of the comments of the reviewer 2 regarding structure of the manuscript, detail missing from the Materials and Methods section, English language and grammar of the manuscript.The manuscript should be improved significantly both from the scientific (clarifying some methodical issues) as well as from technical points of view (English language, gramma) before it can be processed further.

Thank you for your suggestion.In the last round of revision, we have made detailed revisions according to the comments of reviewer 2. In this revision, we have supplemented it. The following are the suggestions put forward by reviewer 2 last time and the modifications we made.

****Note to the authors:****
Entire manuscript needs to be reviewed for edits/structure/grammar. It is not the job of the reviewers or journal staff to edit the manuscript. If the authors cannot complete this task, then they need to hire someone to edit the manuscript. There are too many edits to be listed, therefore the reviewer has only listed select edits as examples:
1. Line 14: Change ‘polymorphism’ to ‘polymorphisms’.

Response: Thank you for your suggestion. We have revised it in the manuscript (Line 14).
2. Line 24: Insert a comma between ‘BW’ and ‘ChW’.

Thank you for your suggestion. We have revised it in the manuscript (Line 31).
3. Line 44: Change ‘cattles’ to ‘cattle’.

Thank you for your suggestion. We have revised it in the manuscript (Line 53).
4. Line 72: Change ‘insulinexpression’ to ‘insulin expression’.

Thank you for your suggestion. We have revised it in the manuscript (Line 90).
5. Line 273: Change “Huir’ to ‘Buir’.
Thank you for your suggestion. We have revised it in the manuscript (Line 342).
6. The authors do not identify the gender of the sheep used in the experiment as gender is included in the model (Line 138).
Thank you for your suggestion. We have revised it in the manuscript (Line 114).
7. Line 152: What is the SNP in exon 7? The authors previously state that 4 exons were evaluated. Assume this is supposed to be SNP7 in exon 4?
Thank you for your suggestion. We have revised it in the manuscript (Line 206).
8. Between the abstract, results, and discussion, the authors are not consistent with the growth traits that are associated with each SNP. The abstract (Line 24-25) states that SNP1 is associated with BW, ChW, and ChC at 4 months and HW at 9 months, and CaC at 4 months. The introduction (Line 81) then states that SNP1 was significantly associated with CaC, ChC, and BW at 4 months and HW at 9 months. Why is ChW not included in the Line 81 statement? It is then stated in Line 179 that SNP1 is associated with WW, ChW, and ChC. Should WW be BW?

Thank you for your suggestion. We have revised it in the manuscript (Line 31-36, 228-234).
9. It is not clear what measurements were made at birth, 4 months, 9 months, and 16 months. Was birth weight the only measurement made at birth?
Thank you for your suggestion. We have revised it in the manuscript (Line 17-21, Line 106-110). At birth, only birth weight was recorded. Body weight, body length, body height, chest circumference, chest depth, chest width, hip width, and cannon circumference were recorded at 4 and 9 months of age respectively; body weight, body length, body height, chest depth, hip width, and cannon circumference were recorded at 16 months of age.
10. Table 4 is confusing as it is labeled for birth and 4 months. Why is there only one measurement for BL, BH, ChW, ChD, ChC, HW, CaC if the table is for 2 time points (birth and 4 months). Time point measurements need to be clarified.
Thank you for your suggestion. We have revised it in the manuscript (Line 17-21, Line 106-110). As the lambs were so small and weak, we only weighed them at birth.
11. Line 179: The authors use the term weaning. What age is weaning?
Thank you for your suggestion. We have revised it in the manuscript (Line 232). Since the time of weaning is not very accurate, we use “4 months of age” instead it.
11. Line 200: The authors state that they recorded productive traits from birth the adulthood. Were all traits measured at birth? This statement is incorrect if body weight was the only trait measured at birth.
Thank you for your suggestion. We have revised it in the manuscript (Line 250-252).
12. The authors conclude the introduction with the statement ‘genetic resources of local sheep breeds of China’. This is an incorrect statement as the authors only evaluated one breed. Delete sentence or revise.
Thank you for your suggestion. We have revised it in the manuscript (Line 97-98).
13. What brand of taq was used for the PCR amplification? As the authors are using the PCR product for sequencing and identification of SNP, it is important to use a high fidelity taq.
Thank you for your suggestion. We have revised it in the manuscript (Line 130).
14. Line 163: The authors state that ‘Ne was calculated”. What is ‘Ne’?
Thank you for your suggestion. We have revised it in the manuscript (Line 216).
15. None of the figures are properly labeled. The linkage disequilibrium plot is labeled at Figure 1 and all the additional figures are not labeled.
Thank you for your suggestion. We have revised them in the manuscript （Fig. 1-3）
16. Overall, the manuscript has serious concerns regarding English/grammar/structure. As a result, the manuscript cannot be properly reviewed. Additionally, there is detail missing from the Materials and Methods section. This has been detailed in the review report.

Thank you for your suggestion. The manuscript has also been double-checked, and the typos and grammar errors we found have been corrected, and changes to our manuscript were all highlighted within the documents by using red colored text. Materials and Methods are also explained and corrected in detail (Line 101-189).
